# Kinematic scattering by nanocrystals

**DOI:** 10.1107/S160057672201069X

**Published:** 2023-02-01

**Authors:** Olivier Thomas, Ismail Cevdet Noyan

**Affiliations:** a Aix Marseille Université, Université de Toulon, CNRS, IM2NP, Marseille, France; b Applied Physics and Applied Mathematics, Columbia University, New York, NY 10027, USA; Montanuniversität Leoben, Austria

**Keywords:** X-ray diffraction, nanocrystals, kinematic scattering

## Abstract

Various formulations are compared which describe diffraction from ultra-thin single-crystal films in the symmetric scattering configuration, showing that, for this thickness range, several implicit assumptions in these formulations are no longer satisfied. Consequently, the position, integrated intensity and integral breadth of a diffraction peak cannot be related to the lattice spacing of the material or the number of unit cells along the diffraction vector using traditional analysis methods. Some simple equations are proposed to obtain the correct values of these parameters for this specific sample/diffraction geometry combination. More generally, the development of rigorous formalisms for analyzing diffraction from nanocrystals is proposed.

## Abstract 2

This work compares various formulations which describe diffraction from ultra-thin single-crystal films and shows that, for this thickness range, several implicit assumptions in these formulations are no longer satisfied. This has important consequences for the analysis of diffraction patterns from nanocrystals.

## 1. Introduction

X-ray diffraction techniques are routinely used for non-destructive characterization of crystalline materials. These methods can be applied to individual crystals or crystalline aggregates and yield quantitative structural and microstructural data from analysis of the position and shape of the relevant Bragg peaks. Structural information such as the symmetry, dimensions and atomic occupancy of the unit cells in the sample are primarily obtained from the angular positions and relative intensities of the Bragg peaks. Any shifts of these Bragg peaks from their ideal positions can be used to compute long-range internal elastic strains and/or composition gradients. Microstructural information such as phase fractions, dimensional parameters (grain size, film thickness), strain and lattice parameter distributions, the presence and concentration of line and planar defects *etc.* is obtained from the intensities, breadths and shapes of the Bragg peaks.

In the past few decades these diffraction techniques have been applied to the characterization of nanocrystalline samples. However, in analogy to the ‘emergent’ properties of nano-solids, some aspects of the scattering process are selectively enhanced on these size scales. This necessitates a careful examination of the canonical formalisms. For example, it was recently shown that, for nanocrystals smaller than 20 nm or so, the classical Lorentz factor and binomial sampling statistics are not applicable, since the multiplicity *m_hkl_* used in these approaches becomes a stochastic parameter for smaller crystallites (Öztürk *et al.*, 2014 ▸, 2015 ▸; Öztürk & Noyan, 2017 ▸). Similarly, it has been known for over a decade that lattice parameters obtained from diffraction analysis of nanoparticles with sizes below 10 nm can deviate significantly from their true values (Bocquet *et al.*, 2003 ▸; Kaszkur *et al.*, 2005 ▸; Kaskur, 2006 ▸; Palosz *et al.*, 2010 ▸), leading to speculation that Bragg’s law was, somehow, not applicable for such sizes; ‘The other important phenomenon observed for nanocrystals is that the diffraction peak positions no longer obey the Bragg law precisely’ (Kaszkur *et al.*, 2005 ▸). Later, Xiong *et al.* (2018 ▸, 2019 ▸) showed that this shift in the Bragg peak positions was due to the increasing influence of the refraction correction with decreasing crystallite size; the shift was more pronounced for smaller Bragg angles and could be substantial for particle dimensions smaller than 5 nm. Since refraction effects are just one of the factors which are size dependent, we undertook the current, broader, study. Here we follow up on the general issue of diffraction from nanocrystals and focus more specifically on the effect of crystallite size on the commonly utilized scattered amplitude formulations at the nanoscale. In this treatment we limit the discussion to an ideally perfect single-crystal thin-film slab diffracting at the kinematic limit, where radial scans through reciprocal points are recorded.

## 2. Theoretical analysis

### 2.1. Description of the problem

**Figure 1:**
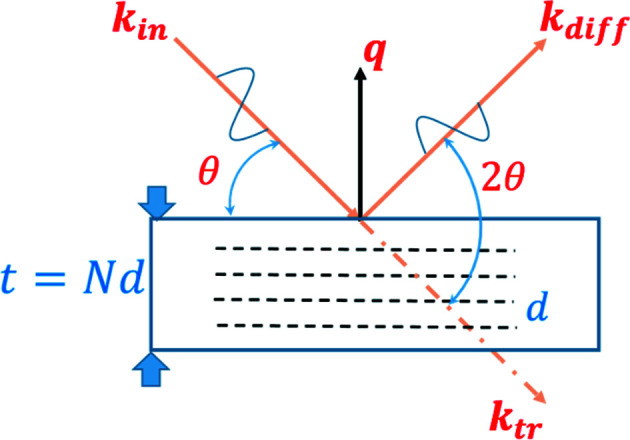
The symmetric diffraction geometry for a thin film of thickness *t* = *Nd*, where *d* is the atomic plane spacing perpendicular to the film surface. The incident, diffracted and transmitted beam vectors **k**_in_, **k**_diff_ and **k**_tr_ are coplanar. The diffraction vector **q** bisects the incident and transmitted beam vectors.

Fig. 1 ▸ depicts the symmetric Bragg diffraction geometry for a single-crystal Si thin-film slab^1^ illuminated with a plane wave of monochromatic X-rays. The largest interplanar distance along the normal to the slab is *d* and any other can be expressed as *d*/*m*, where *m* is an integer. For a stack of *N* planes the total slab thickness is *Nd*. The scattering vector **q** = **k**_diff_ − **k**_in_ is defined as the difference between the incident wavevector and the scattered wavevector. The angle between **k**_diff_ and the transmitted beam vector **k**_tr_ is the diffraction angle 2θ. Thus

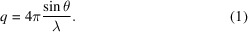




In the case of very thin crystals the total slab thickness will always be much smaller than the extinction length (Authier, 2001 ▸) and thus the kinematic formalism can be used to describe the scattering process. In this framework the scattered amplitude is represented as the Fourier transform of the (triply periodic) electron density within the slab (Patterson, 1939 ▸; Warren, 1990 ▸; Cowley, 1990 ▸),

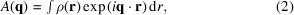

where ρ(**r**) is the electron density distribution function. In the case of an undistorted crystal of finite size, of shape (envelope) function *s*(**r**), the scattered amplitude becomes

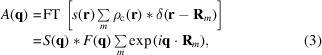

where **R**_*m*_ is a Bravais lattice vector, ρ_c_(**r**) is the electron density in the unit cell, *F*(**q**) is its Fourier transform called the structure factor, *S*(**q**) is the Fourier transform of *s*(**r**), δ is the Dirac delta distribution and the asterisk ( 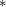 ) represents the convolution product.

In the case of our simple crystal slab diffracting in the radial direction this becomes

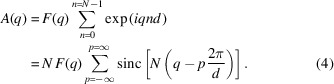

**Figure 2:**
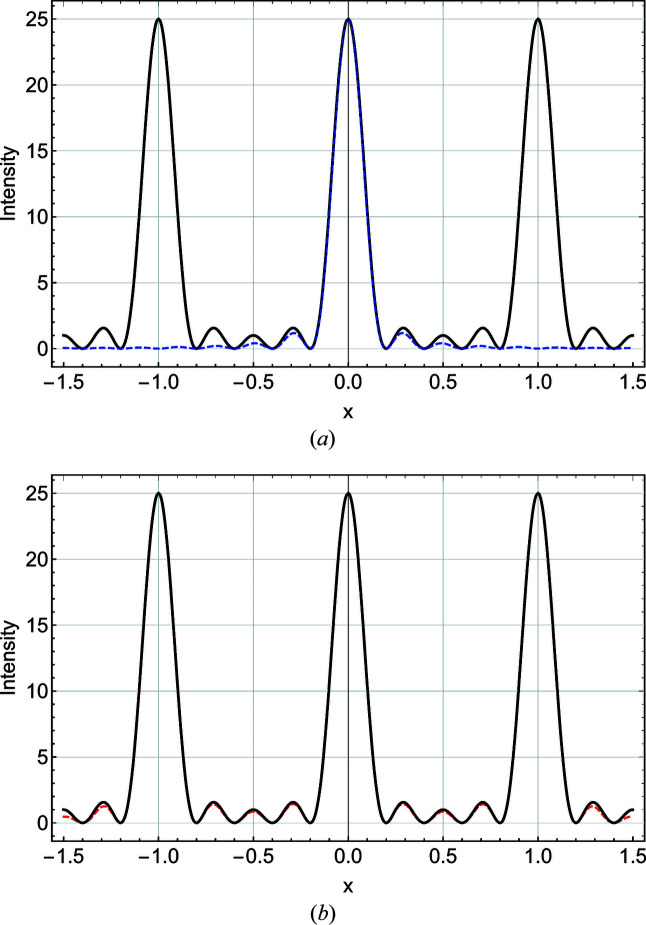
Bragg peak intensities (*N* = 5). (*a*) Laue function *L*(*x*) (black solid line) plotted versus cardinal sine *S*(*x*) (blue dashed line). (*b*) Laue function *L*(*x*) (black solid line) plotted versus the sum of three *S*(*x*) functions centred at *x* = −1, 0 and 1 (red dashed line).

Here the sinc [cardinal sine, sinc(*x*) = sin(*x*)/*x*] arises from the Fourier transform of the slit function. The first sum in (4) is the well known kinematic sum (von Laue, 1948 ▸), which gives rise to a series of Bragg peaks centred at *q*_*p*_ = *p*2π/*d* [Fig. 2 ▸(*a*)]. When *N* decreases the angular acceptance aperture of the crystal broadens, with a concomitant increase in the widths of the series of Bragg peaks. At very large peak breadths most simplifying approximations made in the canonical scattering formulations (Cowley, 1990 ▸) break down. For example, it has already been shown that the refraction correction becomes important for small *N* (Xiong *et al.*, 2018 ▸). Similar considerations might apply for all additional *q*-dependent corrections (Lorentz factor, polarization factor, structure factor *etc.*). Variation of these terms with *q* can modify the shape and, in some cases (depending on the symmetry of the considered function of *q*), the position of the Bragg peaks. Some of these issues are now examined.

If we neglect the *q* dependence of the structure factor, the scattered intensity in the radial direction (**q** perpendicular to the slab, Fig. 1 ▸) from the kinematic sum [equation (4)] can be written as

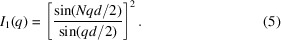


*I*_1_(*q*) is a periodic function whose maxima (Bragg peaks) are at positions

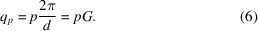




In the following we will focus on the first (*p* = 1) Bragg peak and write

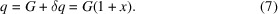

With the reduced dimensionless variable *x* = δ*q*/*G*, *I*_1_(*q*) becomes the Laue function,

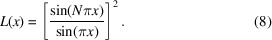




Going back to the general expression of scattered amplitude [equation (4)], one can use the concept of a shape function as developed by Patterson (1939 ▸) and consider – in a completely equivalent way – the scattered amplitude as a sum of sinc functions centred at *q*_*p*_ = *p*2π/*d*. The common approximation, valid for large *N* values, is to consider that a single Bragg peak can be described by a single sinc function. This is not true any more for small values of *N*; here the additional contributions of neighbouring Bragg peaks should also be considered [Fig. 2 ▸(*b*)].

Within the approximation of a single Bragg peak being described by a sinc function, the scattered intensity is proportional to

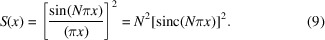




It is common to use expressions (8) and (9) interchangeably in diffraction theory, and they are indeed undistinguishable for large values of *N*, but differences arise for small *N* values (see Fig. 2 ▸).

An important 1/sinθ factor in the scattering amplitude has been discussed by Xiong *et al.* (2018 ▸). The physical origin of this term is derived by Zolotoyabko (2014 ▸) from the summation of scattered waves from a single atomic plane. The equivalence between this scattering description and a refracting description is derived by de Bergevin (1999 ▸) and Als-Nielsen & McMorrow (2011 ▸). Hence in the following this correction factor will be named ‘refraction correction’. The scattered intensities described by equation (8) or (9) do not consider the refraction correction, which introduces a 1/*q* factor in the scattered amplitude (Zolotoyabko, 2014 ▸; Als-Nielsen & McMorrow, 2011 ▸). This results in a 1/*q* ^2^ dependence for the scattered intensity, which can generally be neglected but has important consequences for nanocrystals (Xiong *et al.*, 2018 ▸). The modified Laue function, which includes this refraction correction, becomes

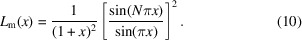




The modified *S*(*x*) function has a similar form:

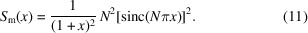




The four intensity distribution functions *L*, *S*, *L*_m_ and *S*_m_ are expressed as functions of the reduced variable *x* = (*q* − *G*)/*G* in reciprocal space. They can be converted to intensity distributions in angular space using the Bragg angle θ_B_, which is defined as

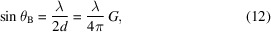

where λ is the wavelength of the incoming radiation.

When this substitution is made, we get *x* = *B*δθ, where *B* = 1/tanθ_B_ and δθ = θ − θ_B_ is the deviation from the Bragg angle.

To summarize, at this point one has four different expressions, equations (8), (9), (10) and (11), for computing or analyzing the radially diffracted intensity (symmetric geometry) from a single-crystal thin-film slab containing *N* planes. Equation (10) is the most accurate form since it considers the refraction correction and is derived directly from the kinematic sum. All the others have implicit assumptions of varying importance. In the following we will compare these four diffracted intensity functions as a function of the number of diffracting planes *N* using numerical simulations, paying special attention to their behaviour for small *N*.

### 2.2. Simulations and analysis

We used *Mathematica 12.0* (Wolfram Research, Champaign, Illinois, USA) to generate intensities with these four functions *L*(*x*), *S*(*x*), *L*_m_(*x*) and *S*_m_(*x*) and fitted them in the range [−4/*N*, 4/*N*]. Following Scherrer (1918 ▸) we used a Gaussian function to fit the primary peaks (Fig. 2 ▸) of these profiles,

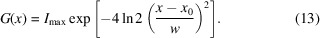

Here *I*_max_, *x*_0_ and *w* are fitting parameters. This function has its maximum value, *I*_max_, at *x* = *x*_0_, with FWHM = *w*, integrated intensity 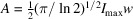 and integral breadth 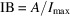 .

We note that, for the normalized sinc function *S*(*x*), *I*_max_ = *N* ^2^, *w* = 0.88589/*N*, *A* = *N* and IB = 1/*N*. Consequently, all values of *I*_max_, *w*, *A* and IB in the following discussion have been normalized by, respectively, *N* ^2^, 1/*N*, *N* and 1/*N*.

### 2.3. Analysis of peak positions

**Figure 3:**
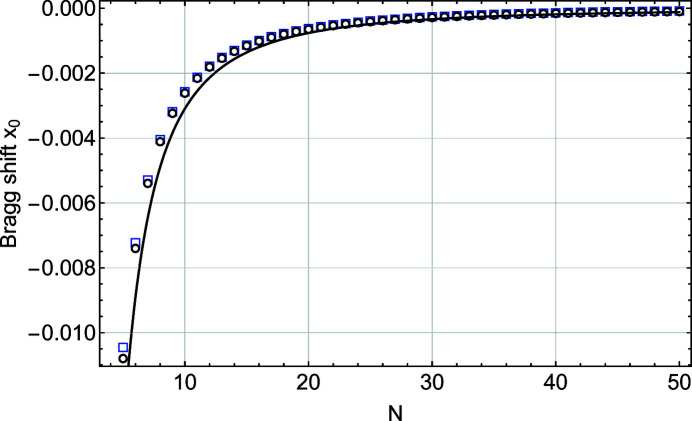
A plot of the Bragg shift *x*_0_ as a function of *N*, the number of planes in the slab. These values are derived from a Gaussian fit to functions *L*_m_(*x*) (black empty circles) and *S*_m_(*x*) (blue empty squares). The solid line is derived from the analytical expression of the position of the maximum of function *L*_m_(*x*) [equation (14)].

As expected, the traditional functions *L*(*x*) and *S*(*x*) are centred at *x*_0_ = 0. On the other hand, the 1/(1 + *x*)^2^ refraction correction slightly skews the functions *L*_m_(*x*) and *S*_m_(*x*) and introduces a finite Bragg shift; *x*_0_ is different from zero for these formulations. This Bragg shift is plotted in Fig. 3 ▸ as a function of *N*.

The Bragg shifts derived for the *L*_m_(*x*) and *S*_m_(*x*) functions are very close to one another. They both show a strong size dependence proportional to 1/*N* ^2^, in agreement with previous work (Xiong *et al.*, 2018 ▸). The position of the maximum of the function *L*_m_(*x*) can be derived analytically,

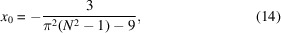

and shows a 1/*N* ^2^ dependency. This is plotted in Fig. 3 ▸ and is in good agreement with the result obtained from a Gaussian fit. The corresponding shifts in angular space, δθ, are easily deduced using *x* = *B*δθ.

To compare the deviations of different Bragg reflections one needs to re-express the slab thickness using the appropriate plane spacing *d_hkl_*; *t* = *Nd* = *N_hkl_d_hkl_*. Then *x*_0_ ∝ −1/*N* ^2^ becomes *x*_0_ ∝ −*d* ^2^/*t* ^2^. This yields the 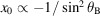 behaviour that has been reported previously (Xiong *et al.*, 2018 ▸).

### 2.4. Analysis of peak intensity and breadth

**Figure 4:**
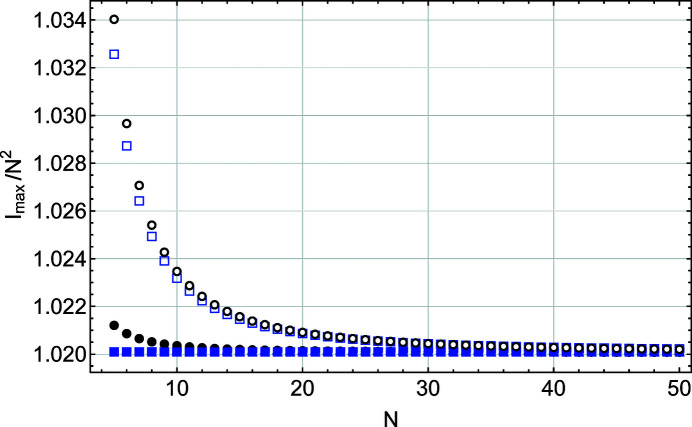
A plot of the maximum intensity scaled by *N* ^2^ as a function of *N*, the number of planes in the slab. These values are derived from a Gaussian fit to functions *L*(*x*) (black filled circles), *S*(*x*) (blue filled squares), *L*_m_(*x*) (black empty circles) and *S*_m_(*x*) (blue empty squares).

Figs. 4 ▸ and 5 ▸ show that, except for the function *S*(*x*), all other parameters (*I*_max_/*N* ^2^, *A*/*N*, *w* × *N* and IB × *N*) obtained from the other three equations exhibit a size dependence, with an increasing departure from the constant value given by the Gaussian fit when *N* decreases. For *I*_max_ (Fig. 4 ▸) the most important offset from the value predicted from *N* ^2^ occurs because of the refraction correction. It varies as 1/*N* ^2^, in agreement with the analytical expectation for the maximum of *L*_m_(*x*),

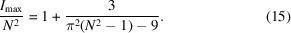




Fig. 4 ▸ also shows that the refraction correction modifies the maximum intensity in the same way for *S*_m_(*x*). The maximum intensity *I*_max_ for the Laue function *L*(*x*) also deviates from the *N* ^2^ prediction at very small *N* values. This deviation is, however, much smaller than the one caused by the refraction correction and might not be important.

**Figure 5:**
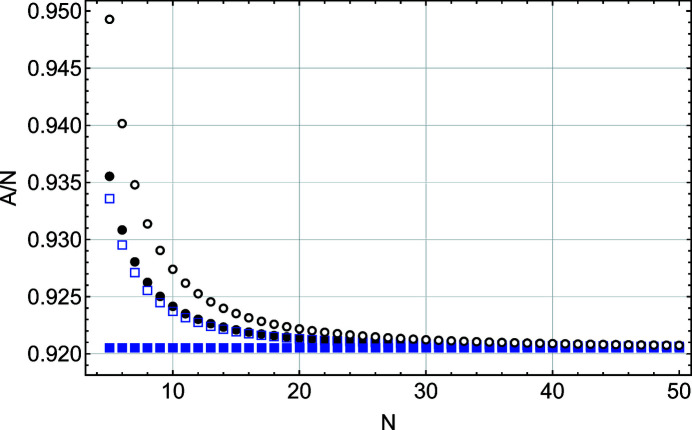
A plot of the integrated intensity scaled by *N* as a function of *N*, the number of planes in the slab. These values are derived from a Gaussian fit to functions *L*(*x*) (black filled circles), *S*(*x*) (blue filled squares), *L*_m_(*x*) (black empty circles) and *S*_m_(*x*) (blue empty squares).

**Figure 6:**
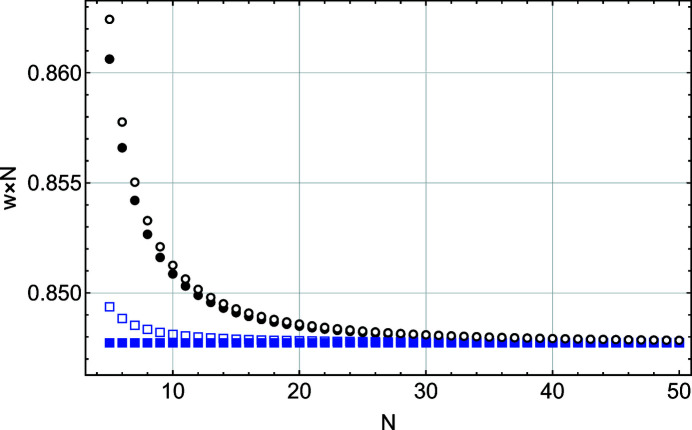
A plot of FWHM scaled by 1/*N* as a function of *N*, the number of planes in the slab. These values are derived from a Gaussian fit to functions *L*(*x*) (black filled circles), *S*(*x*) (blue filled squares), *L*_m_(*x*) (black empty circles) and *S*_m_(*x*) (blue empty squares).

The variation of the integrated intensity *A* with *N* is very different (Fig. 5 ▸). Here *A*, computed from *S*(*x*), is constant for all *N*, while the integrated intensity values obtained from *S*_m_(*x*), *L*(*x*) and *L*_m_(*x*) deviate from the *S*(*x*) values for *N* < 20. The disagreement between the Laue (kinematic sum) and Patterson formulations is even more pronounced when one considers the FWHM and integral breadth behaviours (Figs. 6 ▸ and 7 ▸). To understand this interesting result, we performed a more detailed comparison of the two solutions *S*(*x*) and *L*(*x*).

### 2.5. Comparison of Laue *L*(*x*) and Patterson *S*(*x*) functions

As already mentioned, the parameters of the Patterson function *S*(*x*) are related to the number of planes *N* normal to the diffraction vector **q** with *I*_max_ = *N* ^2^, *w* = 0.88589/*N*, *A* = *N* and IB = 1/*N*. This function is not periodic. On the other hand, the Laue function *L*(*x*) is periodic (with a period of 1; Fig. 2 ▸) and for each maximum *I*_max_ = *N* ^2^, as for *S*(*x*). *L*(*x*) is related to the Fejér kernel (Davis, 1989 ▸) and possesses the interesting normalization property

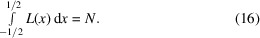

The corresponding equation for *S*(*x*) is

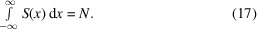

For *L*(*x*), *x* = 1/2 corresponds to the mid-distance between two consecutive Bragg peaks (δ*q* = *G*/2; see Fig. 2 ▸). It is fairly straightforward to compare the integration of *S*(*x*) between −1/2 and 1/2 with that of *L*(*x*), and there is an analytical solution for this comparison:

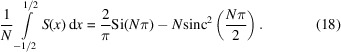

**Figure 8:**
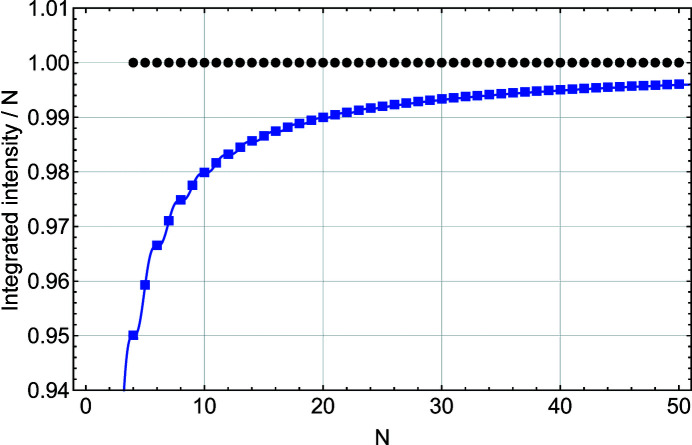
A plot of integrated intensity over the range [−0.5, 0.5] scaled by *N* as a function of *N*, the number of planes in the slab, showing fits to *L*(*x*) (black filled circles) and *S*(*x*) (blue filled squares). The solid blue line corresponds to the analytical prediction [equation (18)]. The analytical prediction for *L*(*x*) is 1 [see equation (16)].

Here Si is the integral cardinal sine (sinc) function. Fig. 8 ▸ shows a comparison of a numerical integration performed between *x* = −1/2 and *x* = 1/2 on *L*(*x*)/*N* and *S*(*x*)/*N*. The analytical result is shown as a solid line. The infinite-*N* limit is unity, showing that a single sinc function is suitable for approximating the Bragg peak for large *N* values. However, a clear deviation between the *S* and *L* functions is observed for small *N*. This indicates that the Bragg peak shape can no longer be described correctly by a single sinc function. To retrieve the correct Laue function, one needs to sum several sinc functions arising from neighbouring Bragg peaks (see Fig. 2 ▸). In other words, the crystal shape function (Patterson, 1939 ▸; Croset, 2017 ▸) can no longer be retrieved from a single Bragg peak, indicating that these solutions are only valid for *N* ≥ 10. For single-crystal silicon films, this corresponds to approximately 5 nm thickness.

**Figure 9:**
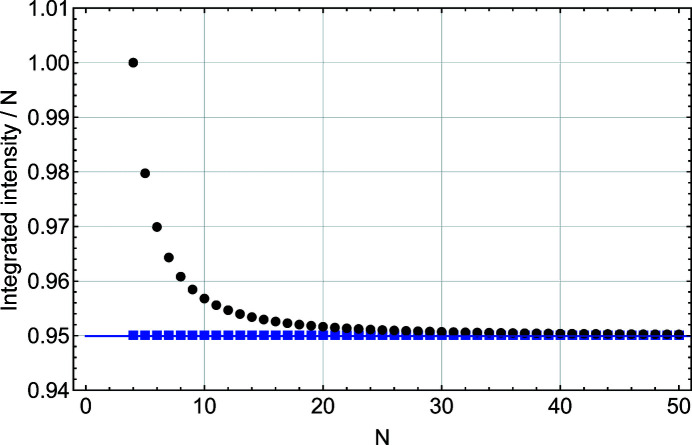
A plot of the integrated intensity over the range [−*r*/2*N*, *r*/2*N*] (with *r* = 4) scaled by *N* (or integral breadth scaled by 1/*N*) as a function of *N*, the number of planes in the slab, showing fits to *L*(*x*) (black filled circles) and *S*(*x*) (blue filled squares). The solid blue line corresponds to the analytical prediction [equation (19)].

**Figure 10:**
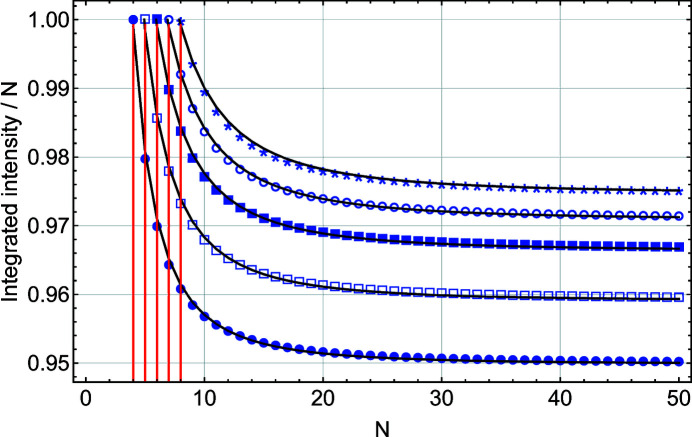
A plot of the integrated intensity for *L*(*x*) over the range [−*r*/2*N*, *r*/2*N*] (with *r* = 4 to 8) scaled by *N* as a function of *N*, the number of planes in the slab. *r* = 4 is shown by filled circles, *r* = 5 by open squares, *r* = 6 by filled squares, *r* = 7 by open circles and *r* = 8 by stars. The vertical red solid lines correspond to *N* = *r*. The solid black lines correspond to the numerical ansatz [equation (21)].

In practice one integrates a diffraction peak on a much smaller range, typically some integer number times the FWHM, usually four if possible. Fig. 9 ▸ shows the result of the integration of *S*(*x*) and *L*(*x*) between −*r*/2*N* and *r*/2*N* with the integer range parameter *r* = 4. The following can be observed:

(i) The integration of *S*(*x*)/*N* in this range yields a constant range-dependent value α(*r*). This agrees with the analytical prediction,

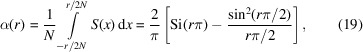

which is independent of *N*.

(ii) In the case of *L*(*x*) the integration range is limited to a single period with *x* = ±½, which in turn limits the integration factor: *r*/*N* ≤ 1. This mathematical limitation would be important in the analysis of synthetic data from ultra-thin films where the magnitudes of *r* and *N* would be comparable (Fig. 10 ▸).

(iii) Finally, we observe that the *L*(*x*) and *S*(*x*) integrated values diverge when *N* < 20. The same behaviour occurs for the integral breadth, since IB is simply the integrated intensity divided by *N* ^2^. Since *L*(*x*) is the exact expression, this has important consequences for the information that can be extracted from the Bragg peak. Because of the necessarily reduced integration range, the integral breadth obtained from *S*(*x*) is no longer proportional to the inverse of the number of diffracting planes (Patterson, 1939 ▸).

To investigate this issue further let us define

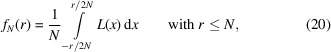

where *f*_*N*_(*r*) has the following properties: *f_N_* (*N*) = 1, *f*_1_(*r*) = *r* and *f*_∞_(*r*) = α(*r*).

In Fig. 10 ▸ *f_N_* (*r*) is plotted for *r* = 4 to 8. In the absence of a known closed-form expression for *f_N_* (*r*) we propose the following simple numerical expression, obtained via least-squares fitting:

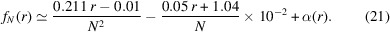

Although a better fit can be obtained using a table of *r*-dependent coefficients, this form gives a reasonable agreement (Fig. 10 ▸).

**Figure 11:**
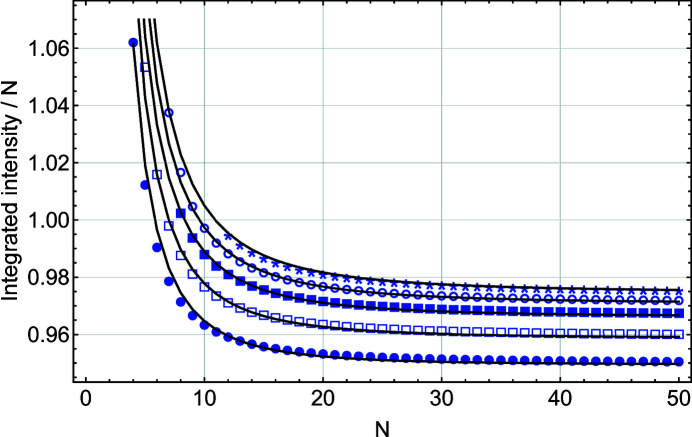
A plot of the integrated intensity for *L*_m_(*x*) over the range [−*r*/2*N*, *r*/2*N*] (with *r* = 4 to 8) scaled by *N* as a function of *N*, the number of planes in the slab. *r* = 4 is shown by filled circles, *r* = 5 by open squares, *r* = 6 by filled squares, *r* = 7 by open circles and *r* = 8 by stars. The solid black lines correspond to the numerical ansatz [equation (23)].

The discussion so far has neglected the effect of the refraction condition on the integrated intensity. Since this correction becomes important for small *N*, we also performed numerical integration of *L*_m_(*x*). Let us define

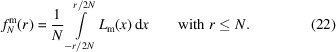




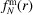
 is plotted in Fig. 11 ▸ for *r* = 4 to 8. The following numerical ansatz, also plotted in Fig. 11 ▸, reproduces the behaviour of the integrated intensity reasonably well:

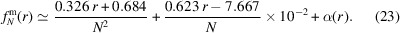

In practice, this expression may be used for linking the integrated intensity or the integral breadth from a nano-film in the range [−*r*/2*N*, *r*/2*N*] to the number of diffracting planes *N*.

## 3. Discussion and conclusions

In this study we have compared the evolution of diffraction peak profiles expected from an ideal single-crystal thin-film sample scattering in the symmetric geometry as a function of film thickness (*N* planes) using four different formulations: (i) kinematic sum [Laue function, equation (8)]; (ii) cardinal sine [shape function of the slab; Patterson function, equation (9)]; (iii) refraction-modified Laue function [equation (10)]; (iv) refraction-modified sinc function [equation (11)]. The following observations are made.

(i) The refraction correction shifts the Bragg peak position towards lower angles, and the amount of this shift varies as 1/*N* ^2^ for both Laue and Patterson formulations. For a given film thickness *N*, the shift is smaller for larger Bragg angles, and varies with 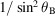 for both approaches. These findings agree with previous reports (Xiong *et al.*, 2018 ▸, 2019 ▸). For our specific sample and diffraction geometry we propose the use of equation (14) to recover the true lattice parameter from experimental or simulated peak profiles.

(ii) The Laue and Patterson formulations yield different peak and integrated intensities and integral breadths for *N* < 20. These differences become larger for decreasing *N*.

(iii) For *N* < 20, the integration of a Bragg peak over a limited angular range (as is the case for experimental data) yields an integrated breadth that is no longer proportional to 1/*N*. We provide a simple ansatz [equation (23)] for correction of this issue. [We note that the corrections described by equations (14) and (23) are strictly valid only for thin films. Similar corrections can be derived for other shapes. However, application of these corrections to nanocrystalline powder diffraction data is non-trivial and requires significant future work, especially if size and shape distributions are present (Xiong *et al.*, 2018 ▸).]

These results show that, when the number of diffracting planes is sufficiently small to cause inordinate increases in the breadths of Bragg peaks, the usual assumption that all *q*-dependent factors (structure factors *etc.*) are sampled at the Bragg peak positions becomes invalid. At this limit these factors can modify the shape of the Bragg peaks, and hence the link between the crystal size and the diffraction peak breadth. For example, it is usual to approximate the Laue function that arises from the scattering of a parallel-faced crystal slab by a single sinc squared function. While this approximation works very well for large enough crystals, it fails at small dimensions (*N* < 20). In this limit the usual Patterson approach (Patterson, 1939 ▸) that assumes that the square modulus of the Fourier transform of the crystal shape function is translated by convolution on each Bragg peak does not work anymore. This has been hinted at by Cowley (1990 ▸, p. 95) and may have important consequences for Bragg coherent diffraction imaging (BCDI) of small crystals. BCDI from Pt crystals as small as 20 nm (this amounts to 88 planes along the 〈111〉 direction) has recently been reported (Richard *et al.*, 2022 ▸). With the continuing increase in coherent flux even smaller crystals will be imaged in the future.

In summary, our work indicates a need for a complete revisiting of the basic concepts of kinematic scattering formulations for nanocrystals, since traditional formulations which connect the position and breadth of Bragg diffraction peaks to the lattice parameters and crystal size of the diffracting crystallites can yield erroneous results.

## Figures and Tables

**Figure 7:**
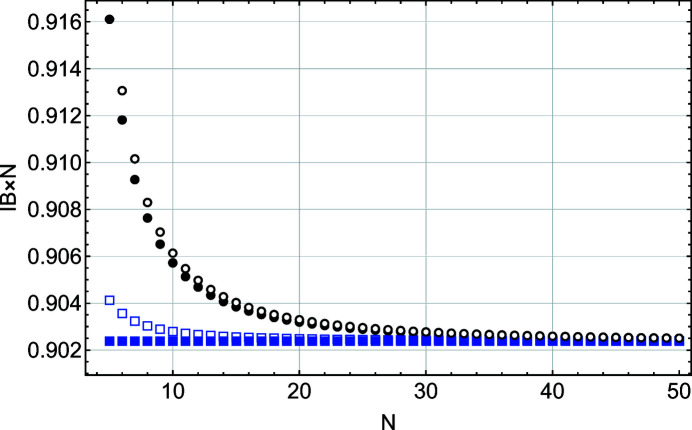
A plot of the integral breadth (IB) scaled by 1/*N* as a function of *N*, the number of planes in the slab. These values are derived from a Gaussian fit to functions *L*(*x*) (black filled circles), *S*(*x*) (blue filled squares), *L*_m_(*x*) (black empty circles) and *S*_m_(*x*) (blue empty squares).

## Footnotes

1 We use Si as an example owing to its technological importance and the availability of very thin silicon on insulator films.
